# Local and Systemic Production of Pro-Inflammatory Eicosanoids Is Inversely Related to Sensitization to Aeroallergens in Patients with Aspirin-Exacerbated Respiratory Disease

**DOI:** 10.3390/jpm12030447

**Published:** 2022-03-11

**Authors:** Daniel P. Potaczek, Gabriela Trąd, Marek Sanak, Holger Garn, Lucyna Mastalerz

**Affiliations:** 1 Translational Inflammation Research Division & Core Facility for Single Cell Multiomics, Medical Faculty, Biochemical Pharmacological Center (BPC), Philipps University of Marburg, 35043 Marburg, Germany; potaczek@staff.uni-marburg.de (D.P.P.); garn@staff.uni-marburg.de (H.G.); 2 2nd Department of Internal Medicine, Jagiellonian University Medical College, 30-688 Krakow, Poland; gabrielatrad@gmail.com (G.T.); nfsanak@cyf-kr.edu.pl (M.S.)

**Keywords:** allergy, aspirin, aspirin-exacerbated respiratory disease (AERD), asthma, atopy, atopic sensitization, eosinophils, eicosanoids, immunoglobulin E, inflammation

## Abstract

Aspirin-exacerbated respiratory disease (AERD) is characterized by overproduction of the pro-inflammatory eicosanoids. Although immunoglobulin E-mediated sensitization to aeroallergens is common among AERD patients, it does not belong to the defining disease characteristics. In this study of 133 AERD patients, we sought to find a relationship between sensitization to aeroallergens and local (leukotriene E_4_, prostaglandin E_2_ and prostaglandin D_2_) and/or systemic (leukotriene E_4_) production of arachidonic acid metabolites. Interestingly, a negative association between pro-inflammatory eicosanoid levels in induced sputum supernatant or urine and sensitization to aeroallergens was observed. This inverse relationship might suggest the presence of a protective effect of atopic sensitization to aeroallergens against stronger local airway inflammation and higher systemic AERD-related inflammatory activity.

## 1. Introduction

Aspirin-exacerbated respiratory disease (AERD) is characterized by asthma, difficult to treat chronic rhinosinusitis with nasal polyps, and acute respiratory reactions precipitated by aspirin or other nonsteroidal anti-inflammatory drugs (NSAIDs) [1,2,3,4,5,6]. Dysregulation of arachidonic acid metabolism leading to the overproduction of the pro-inflammatory eicosanoids, such as leukotriene E_4_ (LTE_4_), by eosinophils, platelet-leukocyte aggregates and mast cells (MCs) is a biochemical hallmark of AERD [7,8,9,10].

Because specific immunoglobulin E (IgE) antibodies to aspirin have not been identified and all the structurally different cyclooxygenase-1 inhibitors cause respiratory reactions in these patients, AERD is not an IgE-mediated allergy, despite the allergic-like reactions to aspirin and NSAIDs that define the disease. On the other hand, atopic sensitization to classical aeroallergens is rather common among AERD individuals, and some AERD patients with and or without a coexisting atopy exhibit higher total serum IgE levels [11,12,13,14,15]. Furthermore, beneficial clinical and biochemical (reduced LTE_4_ production) effects of omalizumab have been observed in AERD subjects [16,17]. Interestingly, omalizumab is a humanized recombinant monoclonal antibody interfering with IgE-dependent MC activation [18,19].

Considering the involvement of IgE-related mechanisms in AERD, we sought, for the first time, to find a relationship between atopic sensitization to aeroallergens and local and systemic eicosanoid production in subjects with AERD.

## 2. Materials and Methods

### 2.1. Patients

This study used a prospective database of one hundred and thirty-three stable AERD patients, who were diagnosed and treated at the 2nd Department of Internal Medicine by Professor Andrzej Szczeklik, Jagiellonian University Medical College, Krakow, Poland. The diagnosis of aspirin hypersensitivity was confirmed by either bronchial or oral aspirin challenge test [20]. The AERD patients were treated with nasal and inhaled corticosteroids, long-acting β2-agonists, and some of them used small doses of oral corticosteroids (*n* = 7) for six weeks preceding the sputum induction. None of the patients used antileukotrienes or biological drugs.

The control group comprised eighty stable asthmatics who tolerated aspirin well (ATA). They did not use aspirin or other NSAIDs for six weeks preceding the study. Otherwise, they were managed the same way as AERD patients.

None of the study participants had a respiratory tract infection or asthma exacerbation in the period of six-week prior to the day of the sputum induction. On that day, every participant had baseline FEV_1_ ≥ 60%. The characteristics of study groups are presented in Table 1. Each of the studies that provided data for this work, and used in this work, received approval from Jagiellonian University Ethics Committee. The study participants gave written informed consent.

### 2.2. Study Design

AERD and ATA patients were recruited on four different occasions (grants), between April 2013 and December 2018 [21,22,23]. The run-in period lasted fifty-two weeks, while patients remained under the ambulatory care of our center. Sputum induction was performed before aspirin challenge. At baseline, before sputum induction, the following evaluations were performed:

(i) clinical evaluation including asthma control test (ACT);
(ii) spirometry;
(iii) skin prick tests;
(iv) inflammatory phenotypes based on induced sputum (IS) cells;
(v) IS supernatant (ISS) eicosanoids (LTE_4_, prostaglandin E_2_—PGE_2_ and prostaglandin D_2_—PGD2);
(vi) peripheral blood eosinophil count;
(vii) total IgE;
(viii) urinary LTE_4_ levels.

### 2.3. Clinical Evaluation

Asthma control was evaluated based on ACT according to GINA 2021 guidelines, where scores of 20–25 correspond to well-controlled asthma, scores of 16–19 to partially controlled, and those of 5–15 to uncontrolled disease [24]. Further, according to GINA 2021 guidelines, asthma severity was assessed retrospectively after at least 2–3 months of treatment, from the level of treatment required to control symptoms and exacerbations. Thereafter, based on this assessment, the patients were classified as those with “mild asthma”—asthma well controlled with step 1 or step 2 treatment, “moderate asthma”—asthma well controlled with step 3 or step 4 treatment, and “severe asthma”—uncontrolled despite optimized treatment, this group corresponded to step 5 treatment [24,25].

### 2.4. Spirometry

Standard spirometry and skin prick tests were performed.

### 2.5. Skin Prick Tests

Skin prick tests for aeroallergens were used to ascertain atopic sensitization status to aeroallergens. A wide panel of aeroallergens (ALK-Abelló, Madrid, Spain), including those most prevalent in our region was used, including the following: house dust mites (*Dermatophagoides pteronyssinus*, *Dermatophagoides farinae*, *Lepidoglyphus destructor*), pollens (*Phleum*, *Lolium*, *Cupressus*, *Platanus*, *Olea*, *Chenopodium*, *Plantago*, *Artemisia*, *Parietaria*, *Salsola kali*), molds (*Alternaria*, *Aspergillus*, *Cladosporium*, *Penicillium*) and animal epithelia (dog, cat, and hamster). Histamine was used as a positive control and saline was used as a negative control. Histamine had to produce a wheal at least three millimeters in diameter. A positive skin prick test to a certain single allergen was defined by a wheal that was equal to or larger than the histamine control [26]. Overall skin prick testing was considered positive and equal to the presence of sensitization to aeroallergens if reactivity to at least one of the aeroallergens was detected.

### 2.6. Induced Sputum Collection

Induced sputum collection was performed according to ERS recommendations [27]. Study subjects inhaled hypertonic saline solution; concentration of solution was increasing from 3% to 5% using ultrasonic nebulizer (Ultraneb 2000; DeVilibiss, Somerset, PA, USA). Induced sputum was manually separated from saliva, after which cytospin slides were obtained. Those were used for differential cell count.

### 2.7. Inflammatory Phenotyping Based on Induced Sputum Cells

Three major inflammatory patterns based on induced sputum cells were differentiated as follows: eosinophilic (≥3% eosinophils and <64% neutrophils), mixed (≥3%, eosinophils and ≥64% neutrophils) and non-eosinophilic (<3% eosinophils) [28,29].

### 2.8. Induced Sputum Supernatant Eoicosanoids

The concentrations of prostaglandin PGE_2_ and PGD_2_ were evaluated in ISS using gas chromatography/mass spectrometry (GC-MS), while those of LTE_4_ were analyzed by high-performance liquid chromatography/tandem mass spectrometry (HPLC-MS/MS). These analytical method details were presented elsewhere [30].

### 2.9. Peripheral Blood Parameters

Total IgE levels in blood were measured by a fluoroenzyme immunosorbent assay (UniCAP; Pharmacia Diagnostics, Uppsala, Sweden).

### 2.10. Urinary Leukotriene E_4_

Samples of urine were collected in the mornings. Enzyme-linked immunosorbent assay (Cayman Chemical Co., Ann Arbor, MI, USA) was used to assess LTE_4_ excretion in urine. Finally, the results were presented in picograms per mg of urinary creatinine.

### 2.11. Statistical Analysis

Categorical variables were presented as numbers and percentages, and analyzed using Pearson’s chi-squared test or Freeman–Halton extension to the Fisher exact test, as appropriate. Continuous variables were tested for the normality of the distribution by Shapiro–Wilk test. Those having normal distribution were expressed as mean ± standard deviation and otherwise as median (interquartile range). For the former, pairwise comparisons were made by Student’s *t*-test, while for the latter by Mann–Whitney U-test. Spearman’s rank correlation coefficient was calculated to assess the relationship between two continuous variables. Multiple linear regression models, including potential confounders as independent predictors, were used to verify the effects of aeroallergen sensitization on local and systemic eicosanoids, with all continuous variables log-transformed before entering the model. A *p*-value of less than 0.05 was considered statistically significant.

## 3. Results

### 3.1. Characteristics of the Study Groups

One hundred and thirty-three patients with stable AERD participated in the study. Their basic demographic, clinical and laboratory characteristics are given in Table 1. Moreover, eighty stable asthmatics tolerating aspirin well (ATA) were included as controls. This group is also characterized in Table 1.

### 3.2. General Correlation Analysis in the Whole AERD Group

**Figure 1:**
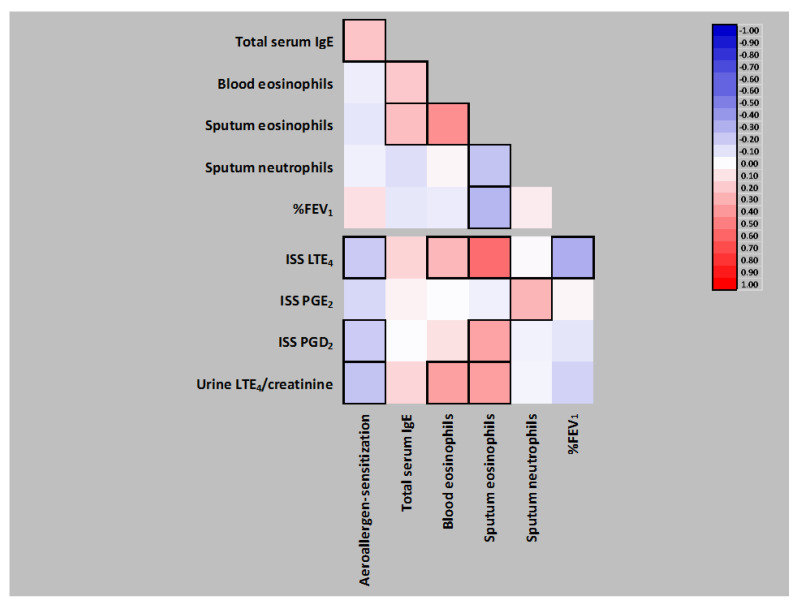
Mutual Spearman’s rank correlations of inflammatory and clinical parameters characterizing our group of 133 patients with aspirin-exacerbated respiratory disease and the correlations of those variables with induced sputum supernatant (ISS) or urine eicosanoids. Significant correlations boxed. IgE, immunoglobulin E; %FEV_1_, % forced expiratory volume in 1 s; LTE_4_, leukotriene E_4_; PGE2, prostaglandin E_2_; PGD2, prostaglandin D_2_.

In the whole AERD group (*n* = 133), % forced expiratory volume in 1 s (%FEV_1_) negatively correlated with IS eosinophils, which in turn positively correlated with blood eosinophil count and inversely with IS neutrophils. Moreover, both blood and IS eosinophils positively correlated with total serum IgE levels (Figure 1). Finally, positive correlations of IS and/or blood eosinophils with ISS LTE_4_, ISS PGD_2_ or urine LTE_4_, a positive correlation of sputum neutrophils with ISS PGE_2_, and an inverse association between urine LTE_4_ and %FEV_1_ were observed (Figure 1).

### 3.3. Analysis of Correlations between Pro-Inflammatory Eicosanoids and Sensitization to Aeroallergens in the Whole AERD Group

Interestingly, in the whole AERD group (*n* = 133), atopic sensitization to aeroallergens inversely correlated with ISS LTE_4_, ISS PGD_2_ and urine LTE_4_ values, despite the presence of an expected positive correlation of aeroallergen sensitization with total serum IgE levels, the latter indirectly linked to ISS LTE_4_, ISS PGD_2_ or urine LTE_4_ levels through the correlation with blood and sputum eosinophils (Figure 1). Total serum IgE levels themselves showed only a tendency (both *p*-values of 0.07) to positively correlate with ISS LTE_4_ or urine LTE_4_ concentrations (Figure 1).

The negative associations of atopic sensitization to aeroallergens with ISS LTE_4_, ISS PGD_2_ or urine LTE_4_ levels remained significant in multiple linear regression models, including atopic sensitization to aeroallergens, total serum IgE concentrations and potential confounders as independent predictors (Table 2). In addition, in the same models, statistical tendencies of total serum IgE levels to positively correlate with ISS LTE_4_ and urine LTE_4_ concentrations observed in univariate analyses became significant (Table 2).

### 3.4. Correlation Analysis in the Control (ATA) Group

In the control group, comprising 80 asthmatics who tolerated aspirin well (ATA), %FEV_1_ negatively correlated with both IS neutrophils and eosinophils. In turn, IS eosinophils positively correlated with blood eosinophil count and total serum IgE levels. Atopic sensitization to aeroallergens positively correlated with total serum IgE concentrations and inversely with IS neutrophils (Supplementary Figure S1).

In addition, serum or IS eosinophils positively correlated with not only ISS LTE_4_, ISS PGD_2_ or urine LTE_4_ levels but also ISS PGE_2_. However, no correlation between ISS or urine eicosanoids and aeroallergen sensitization was observed (Supplementary Figure S1).

### 3.5. Correlation Analysis in a Subgroup of AERD Patients with Eosinophilic Asthma Phenotype

**Figure 2:**
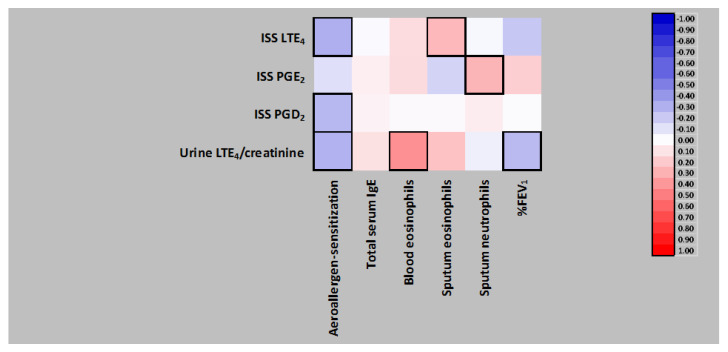
Mutual Spearman’s rank correlations of local and/or systemic eicosanoids and inflammatory or clinical parameters characterizing a subgroup of 61 patients with aspirin-exacerbated respiratory disease having eosinophilic asthma phenotype, and the correlations of those variables with induced sputum supernatant (ISS) or urine eicosanoids. Significant correlations boxed. LTE_4_, leukotriene E_4_; PGE2, prostaglandin E_2_; PGD2, prostaglandin D_2_; IgE, immunoglobulin E; %FEV_1_, % forced expiratory volume in 1 s.

Similarly to the full AERD group, in a subgroup of AERD patients with eosinophilic asthma phenotype (*n* = 61), atopic sensitization to aeroallergens inversely correlated with ISS LTE_4_, ISS PGD_2_ and urine LTE_4_ concentrations (Figure 2). All three associations remained significant in multiple linear regression models, with aeroallergen sensitization, total serum IgE levels and potential confounders as independent predictors (Table 3).

Furthermore, blood eosinophil count positively correlated with IS eosinophils, which negatively correlated with IS neutrophils and %FEV_1_. Blood eosinophils positively correlated with urine LTE_4_, IS eosinophils with ISS urine LTE_4_, and ISS neutrophils with ISS PGE_2_ concentrations. Finally, %FEV_1_ positively correlated with IS neutrophils and negatively with urine LTE_4_ levels (Figure 2 and Supplementary Figure S2).

### 3.6. Correlation Analysis in a Subgroup of AERD Patients with Non-Eosinophilic Asthma Phenotype

**Figure 3:**
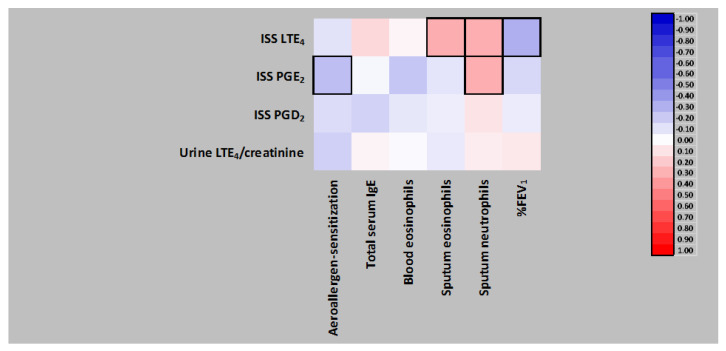
Mutual Spearman’s rank correlations of local and/or systemic eicosanoids and inflammatory or clinical parameters characterizing a subgroup of 66 patients with aspirin-exacerbated respiratory disease having non-eosinophilic asthma phenotype, and the correlations of those variables with induced sputum supernatant (ISS) or urine eicosanoids. Significant correlations boxed. LTE_4_, leukotriene E_4_; PGE2, prostaglandin E_2_; PGD2, prostaglandin D_2_; IgE, immunoglobulin E; %FEV_1_, % forced expiratory volume in 1 s.

In AERD patients with non-eosinophilic asthma phenotype (*n* = 66), only ISS PGE_2_ concentrations inversely correlated with atopic sensitization to aeroallergens (Figure 3), and this association was not resistant to the adjustment of total serum IgE levels and several potential confounders (Table 4). In the same model, a marginally significant association of total serum IgE levels and ISS LTE_4_ concentrations appeared (Table 4).

Furthermore, ISS PGE_2_ levels positively correlated with IS neutrophils, while ISS LTE_4_ concentrations positively with IS neutrophils and eosinophils and negatively with %FEV_1_ (Figure 3 and Supplementary Figure S3).

## 4. Discussion

The major aim of this study was to answer a scientific question as to whether any links between IgE-mediated, atopic sensitization and local and systemic eicosanoid production exist in AERD.

**Figure 4:**
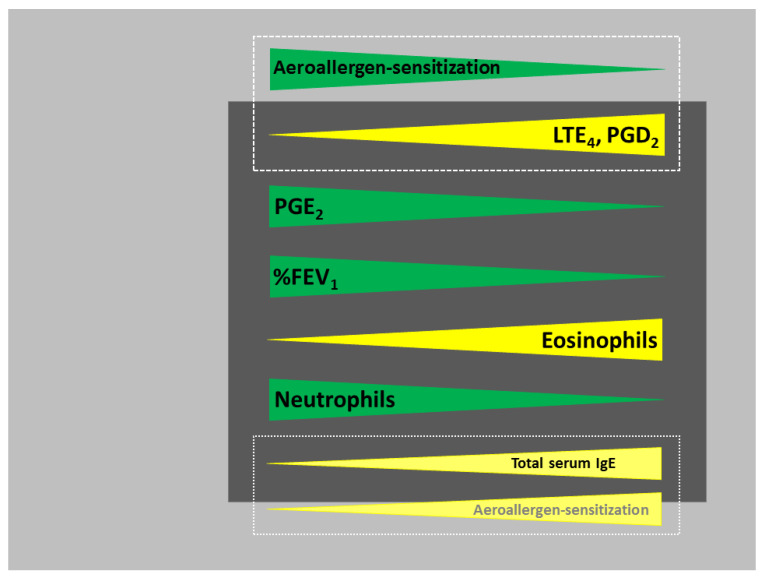
A scheme summarizing the major findings of the study based on the data given in Figure 1 and Table 2: mutual relationships between inflammatory and clinical parameters characterizing our group of 133 patients with aspirin-exacerbated respiratory disease are shown. Eosinophil–neutrophil balance represents a central reference line to which all the other parameters relate to, with only atopic sensitization shown with regard to its relationship to either total serum immunoglobulin E (IgE) or leukotriene E_4_ (LTE_4_)/prostaglandin D_2_ (PGD_2_). Atopic sensitization to aeroallergens inversely correlated with induced sputum supernatant (ISS) LTE_4_, ISS PGD_2_ and urine LTE_4_ values, despite the presence of a positive correlation of aeroallergen sensitization with total serum IgE levels, the latter indirectly linked to ISS LTE_4_, ISS PGD_2_ or urine LTE_4_ levels through the correlation with blood and sputum eosinophils. For details, please refer to the Results, Section 3.2 and Section 3.3, and to the Discussion and Conclusions. %FEV_1_; % forced expiratory volume in 1 s; PGE_2_, prostaglandin E_2_.

However, to validate the generalization potential of the main findings of this investigation, we first analyzed the relationships between the basic pathophysiological parameters of AERD-related inflammation, such as involved immune cells, eicosanoids or IgE, and compared them to the previous observations by other researchers [7,31]. To schematically illustrate this part of our results, shown in more detail in Figure 1 and Table 2, Figure 4 was created, with eosinophil–neutrophil balance representing a central reference line, to which all the other parameters relate.

In brief, a negative correlation between sputum eosinophils and neutrophils was observed as a central pathophysiological feature. While IS eosinophils positively correlated with ISS PGD_2_ and ISS or urine LTE_4_ (major pro-inflammatory arachidonic acid metabolites [32,33]), a similar type of relationship was observed between sputum neutrophils and ISS PGE_2_, a central anti-inflammatory eicosanoid mediator [7,32]. Furthermore, sputum eosinophils negatively correlated with FEV_1_ and thus positively with airway obstruction. Finally, serum blood and sputum eosinophils correlated with each other and total serum IgE levels. Taken together, this part of our results corroborates the observations made by others in similar groups of patients [7,31], suggesting an overall high generalization potential of our findings, including those trying to answer scientific questions asked here for the first time.

In predisposed subjects, aspirin and other NSAIDs cause respiratory symptoms underlain by the allergic-like reactions defining the disorder. However, since no specific IgE antibodies against any of those drugs have been identified, AERD is not an IgE-mediated allergy [11]. Despite substantial progress made in our understanding of the pathobiology of the AERD, neither the triggering cause nor the detailed mechanisms of aspirin/NSAID-induced reactions have been determined. However, although atopy is not a defining feature of the disease, atopic sensitization to aeroallergens and/or related characteristics, such as elevated total serum IgE levels, is common in subjects with AERD, as it is with classic asthmatics [12,13,14,15]. Moreover, elevated levels of total serum IgE have been observed in AERD patients even in the absence of atopy [11]. Further evidence potentially supporting the involvement of IgE-related mechanisms in the pathogenesis of aspirin hypersensitivity has been provided by recent trials on the effectiveness of omalizumab, a humanized recombinant monoclonal antibody interfering with IgE-dependent MC activation [18,19], conducted in patients with AERD. While detailed mechanisms remain to be elucidated [33,34], those studies demonstrated that omalizumab therapy inhibited urinary PGD_2_ and LTE_4_ overproduction, and attenuated upper/lower respiratory tract symptoms during an oral aspirin challenge, resulting in improved aspirin tolerance in the majority of AERD patients treated with omalizumab [16,17].

Thus, and considering that the overproduction of the pro-inflammatory eicosanoids by eosinophils, platelet-leukocyte aggregates and MCs biochemically characterizes AERD [7,8,9,10], we checked whether there is an association between IgE-mediated sensitization to aeroallergens and the local and/or systemic levels of LTE_4_, PGD_2_ and PGE_2_. Surprisingly, opposite to our expectations, urinary and sputum LTE_4_ and sputum PGD_2_, the major pro-inflammatory metabolites of arachidonic acid [32,33], negatively correlated with atopic sensitization to aeroallergens both in crude and adjusted analyzes (Figure 4). Interestingly, this observation might suggest that, in yet an unknown mechanism, the presence of atopic sensitization exerts, in patients with AERD, a kind of protective, local airway inflammation-diminishing and overall disease inflammatory activity-reducing effects. Moreover, despite a positive correlation between total serum IgE levels and atopic sensitization to aeroallergens, statistical tendencies of total serum IgE levels to positively correlate with ISS LTE_4_ and urine LTE_4_ observed in crude models became significant in multivariate analyzes. This finding might, in turn, suggest the presence of a dual, bidirectional relationship between IgE system and AERD-related local and systemic inflammation. In this scenario, aeroallergen-specific IgE molecules, corresponding to the presence of atopic sensitization, would protect, whereas allergen-non-specific, polyclonal IgE, reflected by total serum IgE mass, would either contribute to or, most probably, be only an epiphenomenon related to AERD-related inflammation and/or stimulation by other factors, such as staphylococcal enterotoxins [35]. Even though the presence of atopic sensitization and total serum IgE levels obviously correlate, different patterns of the associations they show with other traits, such as eicosanoids, cannot be surprising due to many possible reasons. For example, it is mostly varying genetic mechanisms that influence total and specific IgE production [36,37], and it has been shown that specific serum IgE against certain allergens can account for up to 20–25% of total serum IgE, driving the overall value of total blood serum IgE [38]. Furthermore, as partially mentioned above, even though total serum IgE levels are known to correlate with the presence of asthma or its severity, also independently of atopy [39,40], it is not fully elucidated if they mechanistically contribute to or are only a secondary epiphenomenon of asthma [36,37], possibly resulting from a polyclonal stimulation by other factors, e.g., staphylococcal enterotoxins [35].

While eosinophilic inflammation in upper and lower airways, together with MC activation, play a key role in the pathogenesis of AERD [23,41,42], non-eosinophilic airway inflammation-related mechanisms may also contribute in some AERD patients [22,43,44,45,46]. Therefore, we performed our analyzes also after substratification of AERD individuals according to the IS cytology-determined type of airway inflammation. Interestingly, similarly to the whole AERD group, but with a stronger magnitude, atopic sensitization to aeroallergens was found in AERD patients with eosinophilic asthma inversely associated with urinary and sputum LTE_4_ and sputum PGD_2_. On the contrary, no robust association between arachidonic acid metabolites and atopic sensitization was observed in the subgroup of AERD patients with non-eosinophilic airway inflammation; the only correlation observed for ISS PGE_2_ in a crude analysis disappeared when tested in a multivariable model. Thus, the effect observed in the whole AERD group was probably driven by the subgroup of patients with eosinophilic asthma.

Our study has several strengths and weaknesses. The major strengths include, for AERD, a unique analysis of IS cytology and ISS eicosanoids, and a comparatively high number of well-characterized AERD subjects included in the study. The major limitation is a single time-point analysis, while the longitudinal design would make it possible to determine a predictive effect of sensitization to aeroallergens at the baseline for the levels of local and systemic eicosanoids during the follow-up.

## 5. Conclusions

In summary, to the best of our knowledge for the first time, we studied and observed the total serum IgE-independent reverse association between the presence of atopic sensitization to aeroallergens and the levels of major local and systemic pro-inflammatory arachidonic acid metabolites in AERD patients. Our results might suggest the presence of the protective effect of atopic sensitization to aeroallergens against stronger local (airway) and overall/systemic AERD-related inflammation. However, further studies, e.g., those on the interplay between systemic and local IgE, including specificities directed against some nasal bacteria antigens [6,47], are required to fully elucidate the mechanisms underlying our observation. In addition, whether our findings could translate into clinical effects, e.g., those on disease severity, should be investigated in larger clinical studies.

## Figures and Tables

**Table 1:** Characteristics of the major and control study groups.

	Aspirin-Exacerbated Respiratory Disease (*n* = 133)	Aspirin-Tolerant Asthma (*n* = 80)
Age, years	49.0 (39.0–55.0)	48.0 (38.0–57.0)
Males, *n* (%)	38 (28.6)	38 (47.5)
Body mass index, kg/m^2^	26.4 (23.7–29.8) (*n* = 132)	25.7 (24.1–29.0)
Disease duration, years	10.0 (6.0–17.0)	11.5 (5.0–20.5)
Age of disease onset, years	35.0 (26.0–43.0)	36.0 (20.5–46.5)
Forced expiratory volume in 1 s, %	90.1 ± 15.7	95.1 ± 16.1
Chronic sinusitis, *n* (%)	133 (100.0)	43 (53.8)
ACT score	22.0 (18.0–25.0)	23.0 (20.0–25.0)
Asthma control level		
Controlled, *n* (%)	88 (66.2)	64 (80.0)
Partly controlled, *n* (%)	27 (20.3)	10 (12.5)
Not controlled, *n* (%)	18 (13.5)	6 (7.5)
GINA severity asthma level		
Mild, *n* (%)	18 (13.5)	29 (36.3)
Moderate, *n* (%)	17 (12.8)	9 (11.3)
Severe, (%)	98 (73.7)	42 (52.5)
Corticosteroids (CS)		
Inhaled CS, *n* (%)	121 (91.0)	71 (88.8)
Inhaled CS dose (fluticasone propionate or equivalent), mg	500 (250–1000)	400 (113–1000)
Oral CS, *n* (%)	7 (5.3)	7 (8.8)
Blood eosinophils, cells/mm^3^	350 (220–548)	297 (153–515)
Total serum immunoglobulin E, IU/mL	101.0 (35.9–224.0)	98.7 (35.1–259.0)
Positive skin prick testing, *n* (%)	48 (36.1)	52 (65.0)
Induced sputum (IS) cytology		
Neutrophils, %	39.5 (20.5–56.5)	47.6 (33.4–62.8)
Eosinophils, %	3.0 (0.6–11.3)	1.1 (0.0–3.1)
IS cytology-based asthma phenotype		
Eosinophilic, *n* (%)	61 (45.9)	18 (22.5)
Non-eosinophilic, *n* (%)	66 (49.6)	59 (73.8)
Mixed, *n* (%)	6 (4.5)	3 (3.8)
IS supernatant eicosanoids		
Leukotriene E_4_, pg/mL	43.0 (12.6–131.9) (*n* = 128)	17.5 (6.7–43.6) (*n* = 79)
Prostaglandin E_2_, pg/mL	58.3 (37.5–100.3) (*n* = 131)	51.5 (39.0–90.1)
Prostaglandin D_2_, pg/mL	34.1 (17.0–88.0) (*n* = 132)	23.9 (13.9–62.3) (*n* = 79)
Urinary leukotriene E_4_/creatinine, pg/mg	1050 (510–2362)	358 (130–812)

Continuous data are given as mean ± standard deviation or median (interquartile range), as appropriate.

**Table 2:** Multiple regression models verifying the effects of aeroallergen sensitization on local and systemic eicosanoids in the whole group of patients with aspirin-exacerbated respiratory disease (*n* = 133).

Independent Predictors Included in Multivariate Models	Induced Sputum Supernatant Leukotriene E_4_ *		Induced Sputum Supernatant Prostaglandin E_2_ *		Induced Sputum Supernatant Prostaglandin D_2_ *		Urine Leukotriene E_4_/Creatinine *	
	β	*p*-Value	B	*p*-Value	B	*p*-Value	β	*p*-Value
Positive skin prick testing	−0.27	0.003	−0.13	0.16	−0.21	0.02	−0.30	0.001
Total serum immunoglobulin E *	0.23	0.01	0.04	0.64	0.03	0.73	0.25	0.006
Age *	−0.10	0.28	−0.06	0.55	−0.11	0.25	0.02	0.85
Male sex	0.12	0.18	0.19	0.04	0.04	0.64	0.09	0.27
Body mass index *	0.10	0.28	0.07	0.47	0.09	0.32	0.01	0.88
Inhaled corticosteroids	−0.06	0.53	−0.04	0.66	−0.02	0.85	0.04	0.66
Oral corticosteroids	−0.05	0.56	−0.07	0.44	−0.13	0.14	0.03	0.69

* All continuous variable log-transformed before entering the models.

**Table 3:** Multiple regression models verifying the effects of aeroallergen sensitization on local and systemic eicosanoids in aspirin-exacerbated respiratory disease patients with eosinophilic asthma phenotype (*n* = 61).

Independent Predictors Included in Multivariate Models	Induced Sputum Supernatant Leukotriene E_4_ *		Induced Sputum Supernatant Prostaglandin E_2_ *		Induced Sputum Supernatant Prostaglandin D_2_ *		Urine Leukotriene E_4_/Creatinine *	
	β	*p*-Value	B	*p*-Value	B	*p*-Value	β	*p*-Value
Positive skin prick testing	−0.36	0.01	−0.07	0.59	−0.37	0.007	−0.45	<0.001
Total serum immunoglobulin E *	0.03	0.86	0.01	0.97	0.09	0.52	0.15	0.21
Age *	−0.13	0.36	0.04	0.76	0.02	0.87	−0.19	0.12
Male sex	0.16	0.27	0.32	0.03	0.11	0.40	0.27	0.03
Body mass index *	0.13	0.38	−0.02	0.91	0.22	0.12	0.14	0.25
Inhaled corticosteroids	−0.05	0.73	−0.01	0.92	−0.09	0.48	−0.03	0.80
Oral corticosteroids	0.01	0.96	−0.26	0.06	−0.09	0.50	0.25	0.04

* All continuous variables log-transformed before entering the models.

**Table 4:** Multiple regression models verifying the effects of aeroallergen sensitization on local and systemic eicosanoids in aspirin-exacerbated respiratory disease patients with non-eosinophilic asthma phenotype (*n* = 66).

Independent Predictors Included in Multivariate Models	Induced Sputum Supernatant Leukotriene E_4_ *		Induced Sputum Supernatant Prostaglandin E_2_ *		Induced Sputum Supernatant Prostaglandin D_2_ *		Urine Leukotriene E_4_/Creatinine *	
	β	*p*-Value	B	*p*-Value	B	*p*-Value	β	*p*-Value
Positive skin prick testing	−0.13	0.33	−0.18	0.19	−0.09	0.47	−0.21	0.13
Total serum immunoglobulin E *	0.27	0.04	0.02	0.89	−0.22	0.09	0.05	0.73
Age *	−0.17	0.21	−0.11	0.43	−0.20	0.15	0.19	0.19
Male sex	0.29	0.03	0.06	0.64	0.03	0.79	−0.11	0.41
Body mass index *	0.09	0.52	0.20	0.18	−0.07	0.61	−0.05	0.74
Inhaled corticosteroids	−0.12	0.37	−0.06	0.64	0.12	0.35	0.07	0.57
Oral corticosteroids	0.04	0.74	0.04	0.77	−0.19	0.14	−0.05	0.73

* All continuous variables log-transformed before entering the models.

## Data Availability

The data presented in this work are available on request from the corresponding author.

## Supplementary Materials

The following supporting information can be downloaded at: https://www.mdpi.com/article/10.3390/jpm12030447/s1, Figure S1: Mutual Spearman’s rank correlations of inflammatory and clinical parameters characterizing our control group of 80 patients with aspirin-tolerant asthma and the correlations of those variables with induced sputum supernatant (ISS) or urine eicosanoids, Figure S2. Full set of mutual Spearman’s rank correlations of inflammatory and clinical parameters characterizing a subgroup of 61 patients with aspirin-exacerbated respiratory disease having eosinophilic asthma phenotype and the correlations of those variables with induced sputum supernatant (ISS) or urine eicosanoids, Figure S3. Full set of mutual Spearman’s rank correlations of inflammatory and clinical parameters characterizing a subgroup of 66 patients with aspirin-exacerbated respiratory disease having non-eosinophilic asthma phenotype and the correlations of those variables with induced sputum supernatant (ISS) or urine eicosanoids.
