# Supplementary material for: Local and Systemic Production of Pro-Inflammatory Eicosanoids Is Inversely Related to Sensitization to Aeroallergens in Patients with Aspirin-Exacerbated Respiratory Disease
Source: J Pers Med. 2022 Mar 11;12(3):447. doi: 10.3390/jpm12030447 (PMC8955638; doi:10.3390/jpm12030447)
Supplement: Supplementary file 1 [file jpm-12-00447-s001.zip › jpm-1619924-supplementary.pdf]

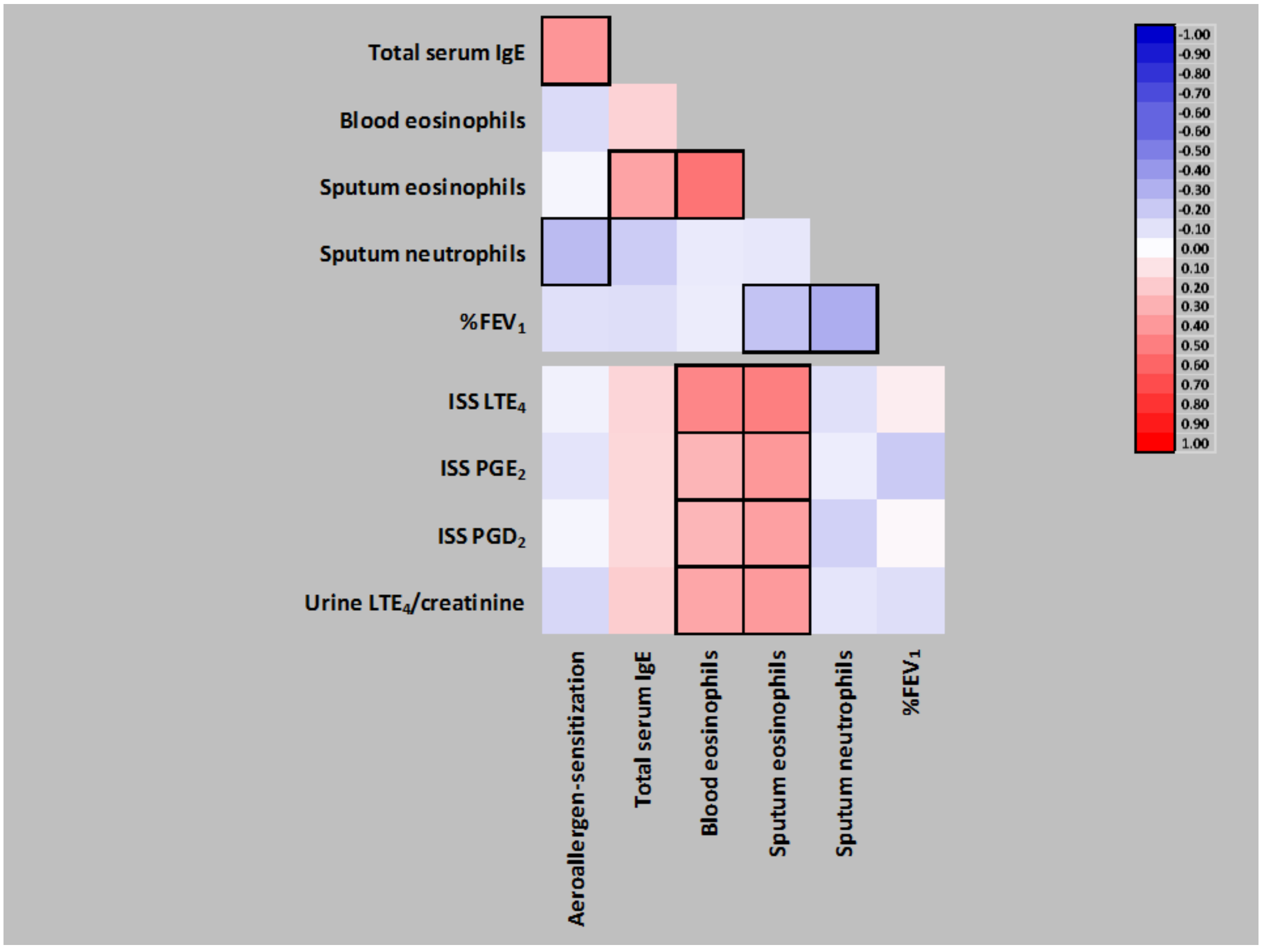

Figure S1. Mutual Spearman's rank correlations of inflammatory and clinical parameters characterizing our control group of 80 patients with aspirin-tolerant asthma and the correlations of those variables with induced sputum supernatant (ISS) or urine eicosanoids. Significant correlations boxed. IgE, immunoglobulin E; %FEV_1, % forced expiratory volume in 1 second; LTE_4, leukotriene E_4; PGE2, prostaglandin E_2; PGD2, prostaglandin D_2.

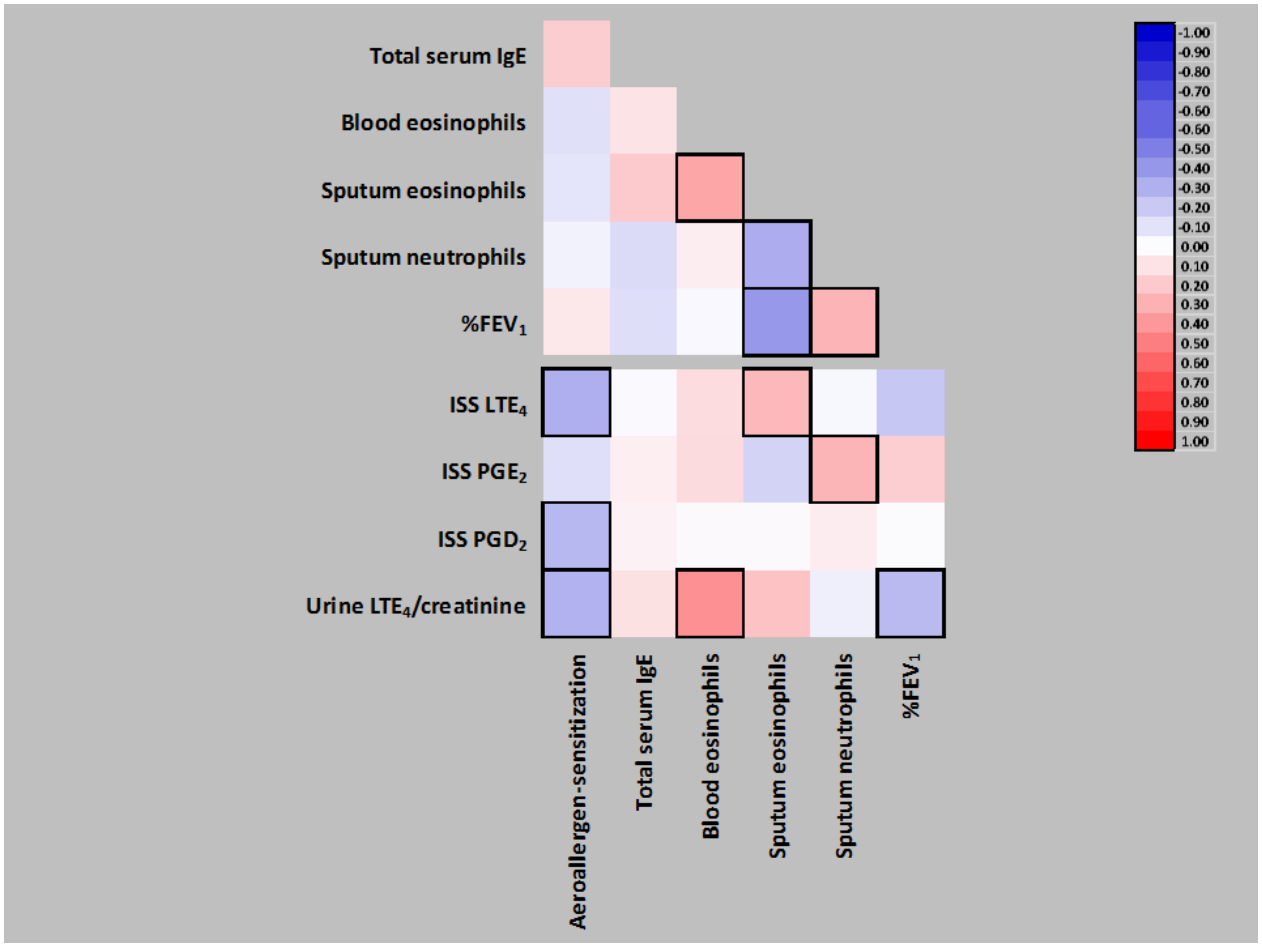

Figure S2. Full set of mutual Spearman's rank correlations of inflammatory and clinical parameters characterizing a subgroup of 61 patients with aspirin-exacerbated respiratory disease having eosinophilic asthma phenotype and the correlations of those variables with induced sputum supernatant (ISS) or urine eicosanoids. Significant correlations boxed. IgE, immunoglobulin E; %FEV_1, % forced expiratory volume in 1 second; LTE_4, leukotriene E_4; PGE2, prostaglandin E_2; PGD2, prostaglandin D_2.

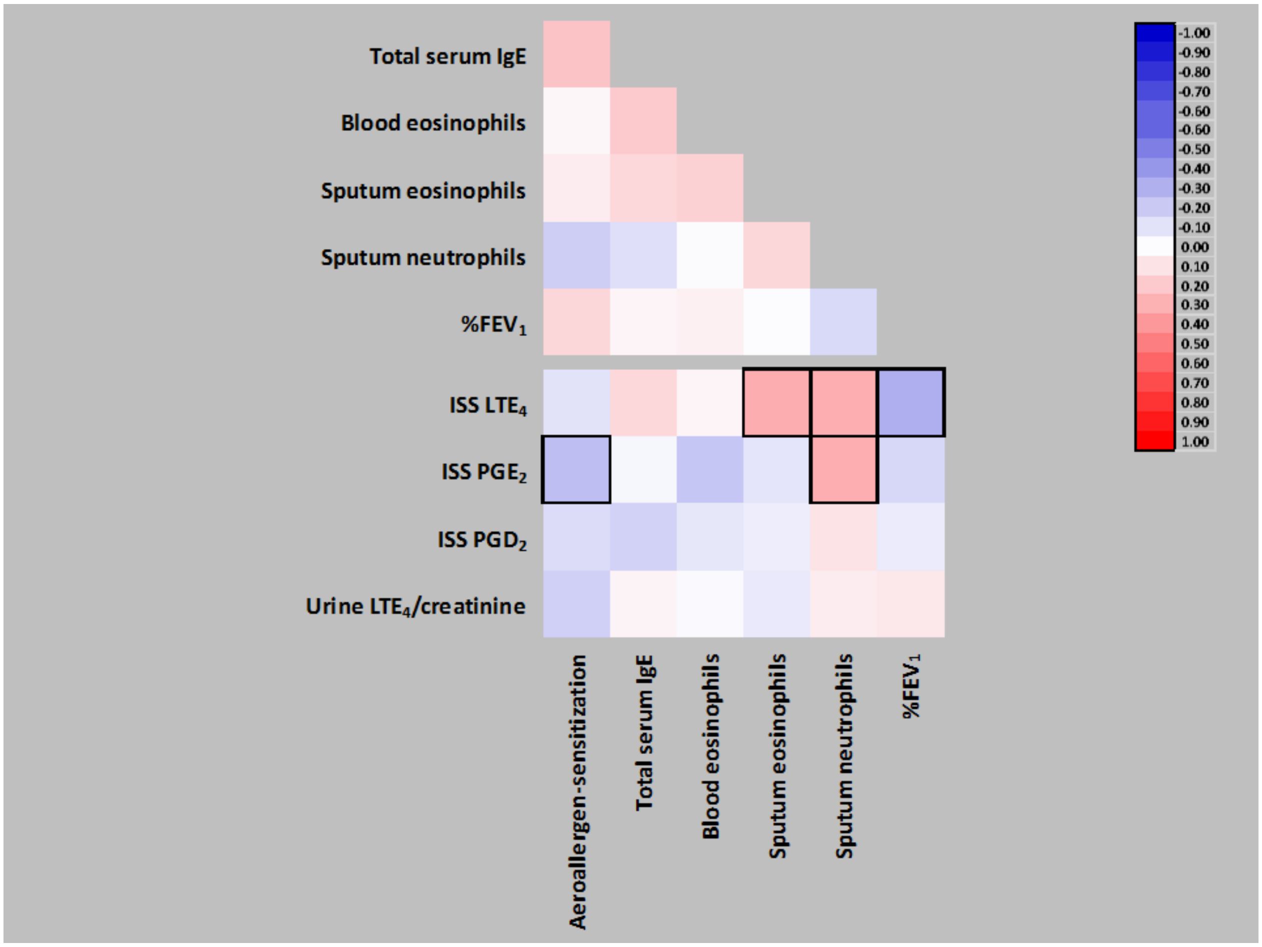

Figure S3. Full set of mutual Spearman's rank correlations of inflammatory and clinical parameters characterizing a subgroup of 66 patients with aspirin-exacerbated respiratory disease having non-eosinophilic asthma phenotype and the correlations of those variables with induced sputum supernatant (ISS) or urine eicosanoids. Significant correlations boxed. IgE, immunoglobulin E; $\%FEV_1$, % forced expiratory volume in 1 second; LTE_4, leukotriene E_4; PGE2, prostaglandin E_2; PGD2, prostaglandin D_2.
